# Recurrent Stress-Induced Cardiomyopathy: A Case Report and Review Article

**DOI:** 10.1155/2011/160802

**Published:** 2011-10-17

**Authors:** Suman krishna Kotla, Cyril Nathaniel

**Affiliations:** ^1^Department of Internal Medicine, Memorial Medical Center, 1086 Franklin street, Johnstown, PA 15905, USA; ^2^Department of Cardiology, Memorial Medical Center, 1086 Franklin street, Johnstown, PA 15905, USA

## Abstract

ABS is a unique acute cardiac syndrome and a recently recognized form of transient left ventricular dysfunction. It mimics ACS in clinical presentation (chest pain and dyspnea) and specific ECHO findings in the absence of significant coronary lesions. This rare entity accounts for 2.2% of ST segment elevation ACS. Pathophysiology mostly correlates to stress-induced catecholamine release. The syndrome is predominant in females, mostly in the postmenopausal age group. It should be initially managed according to the guidelines of ACS. The prognosis for apical ballooning syndrome is generally favorable with inpatient hospital mortality less than 2%. Reports of a single episode of ABS are common in recent medical literature; we report a rare case of recurrence that provides more insight into the nature of this unique syndrome.

## 1. Introduction

Stress cardiomyopathy is a syndrome of transient cardiac dysfunction precipitated by intense emotional or physical stress. A linear relationship between emotional/physical stress and adverse cardiovascular events such as death and MI has been recognized for many years [1, 2]. Wantanabe et al. reported acute cardiac events after an earthquake in Niigata, Japan, on October 23, 2004. Similarly, neurologists recognized an association between subarachnoid hemorrhage and reversible cardiomyopathy that has been described as neurogenic stunning, characterized by acute brain injury and the absence of CAD [3–5]. 

In the early 1990s, Japanese physicians described a clinical entity that has been described over the years as Apical Ballooning Syndrome/Stress-Ampulla Cardiomyopathy/Tako-Tsubo Cardiomyopathy/Transient Left Ventricular Ballooning Syndrome/Broken Heart Syndrome/Cardiac Syndrome X. Over the past decade, several case reports, multicenter retrospective analyses, and systemic reviews of this novel, yet well-described, reversible cardiomyopathy have been published, but much remains unknown about this entity. Recurrence of this syndrome is rare and infrequent. In the largest series so far, consisting of 88 patients, a recurrence rate of 2.7% has been reported [6]. We present a rare case of recurrent stress-induced cardiomyopathy.

## 2. Case Presentation

**Figure 1:**
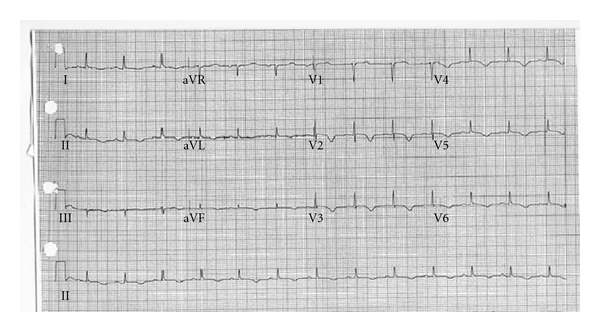
EKG: sinus rhythm and normal axis, significant for deep anterolateral T wave inversions.

**Figure 2:**
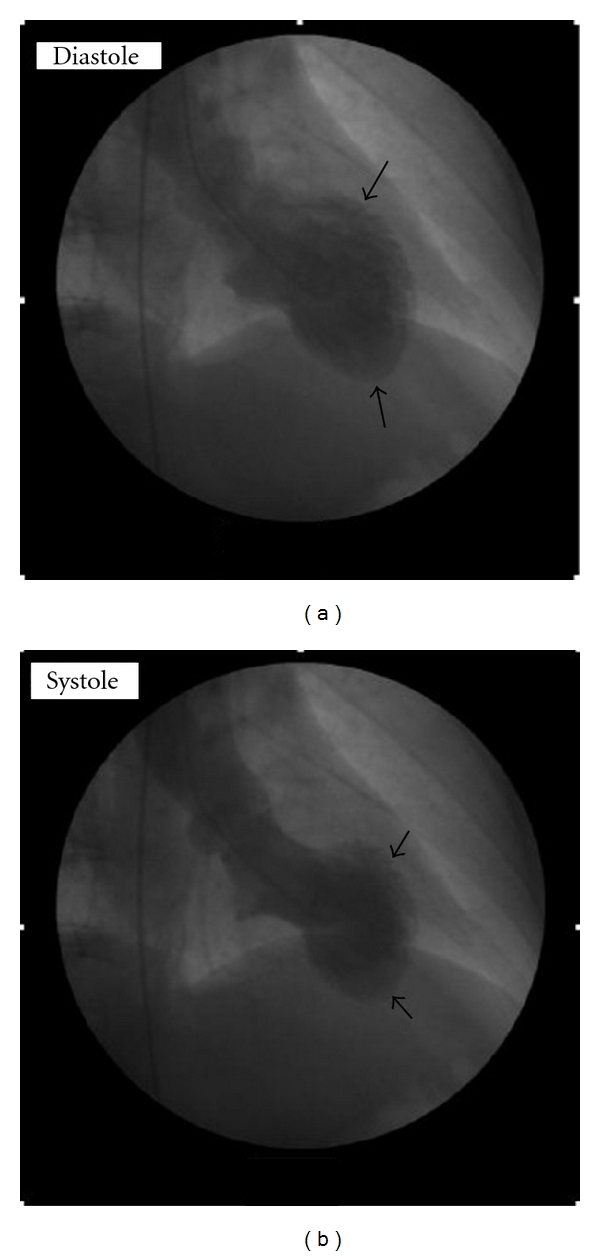
Diastolic and systolic frames from left ventriculography illustrating akinesis of mid and apical segments.

**Figure 3:**
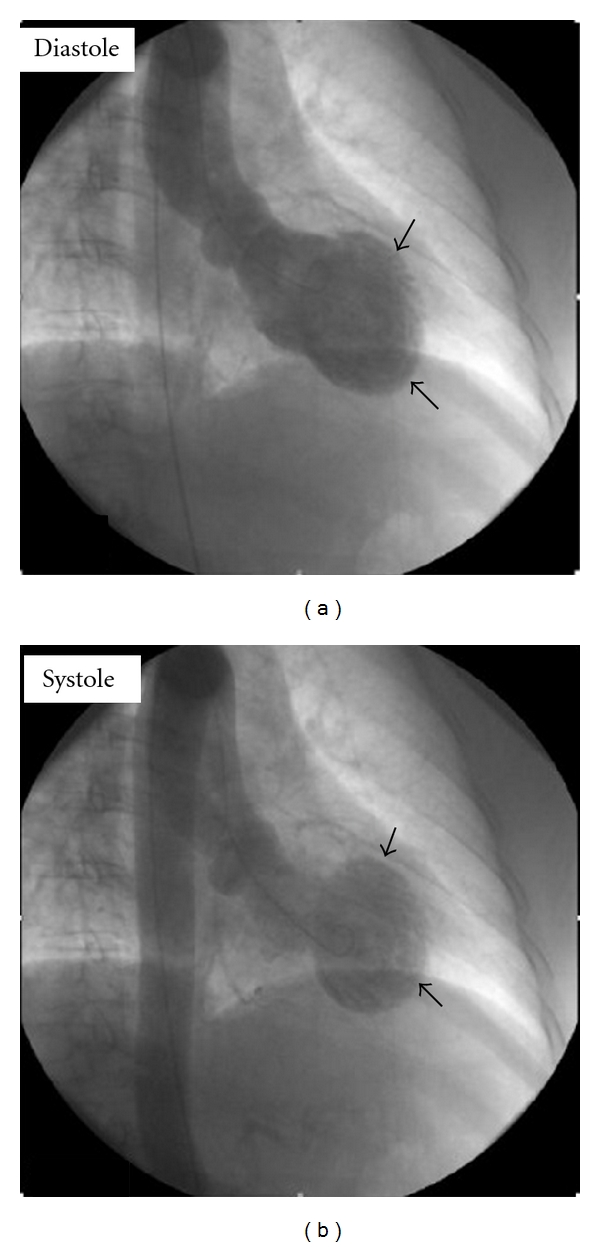
Diastolic and systolic frames from left ventriculography of a patient with akinesis of midsegment and normal apical contraction (recurrent stress-induced cardiomyopathy after a gap of 5 years).

A 64-year-old female with past medical history significant for HTN, DM type 2, arthritis, and a family history of premature coronary disease presented to the ER with chest pain after learning of her mother's death. Pain was mainly retrosternal, continuous, graded 7/10, with no aggravating or relieving factors, and no change in position or respiration. Physical examination was normal. An EKG obtained was significant for T-wave inversions in precordial leads (Figure 1), positive for Troponin I 5.3 ng/mL (normal < 0.01), and with a peak CKMB of 8.5 ng/mL (which is upper limit of normal (0–6.6)). The patient was started on nitro, beta-blocker, and heparin treatment. The coronary angiography performed was insignificant for any anatomical lesion, and ventriculography was significant for severe hypokinesis of anterolateral, apical, and apical septal regions, with an ejection fraction of 49% (Figures 2(a) and 2(b)). Myocardial biopsy did not reveal any inflammation. The absence of any detectable lesion was unexpected in this patient, with deep anterolateral T-wave inversions and positive troponin. It was hypothesized that the sudden grief reaction after learning of the death of her elderly mother might have caused a sympathetic surge, LV stunning anteriorly, and evolving deep T-wave inversions without Q-waves. The patient was discharged on ASA 81 mg, Plavix 75 mg, and a beta-blocker (Lopressor 25 mg BID); a follow-up ECHO at 3 months showed LVEF of 59%. And the patient was recommended to be off from Plavix and beta-blocker since the EF was back to normal. Four years later, on learning about the sudden unexpected death of her brother, she developed left-side chest pain. This started around 9:30 in the morning; the patient presented to the ER at 10:06 AM. In the ER, the patient was found to be tachycardic; pain was continuous, graded 8/10, and nonradiating, with no aggravating or relieving factors. Physical examination was normal. An EKG obtained was significant for sinus tachycardia with a rate of 112, nondiagnostic Q-waves in septal leads and hyperacute peak T-waves in lead v2 (hyperacute T-waves could represent an early stage of MI), troponin I of 0.56 ng/mL. Coronary angiography was insignificant for any lesion, and ventriculography was significant for akinesis of the Ant-lat region with LVEF of 29% (Figures 3(a) and 3(b)). The myocardial biopsy was normal. The patient was discharged in stable condition on ASA, beta-blocker, and ACEI (lisinopril 5 mg po daily). A follow-up ECHO at 6 months showed an EF of 58% with no wall-motion abnormalities.

## 3. Discussion

In 2006, AHA incorporated ABS into the classification of cardiomyopathies as a primary acquired cardiomyopathy [7]. The incidence of ABS as per the case reports and literature review is apparently 1.5%–2.2% in patients presenting with suspected anterior wall MI [6, 8]. According to AHA statistics, there are six to seven million hospital discharges with a primary diagnosis of acute MI in the USA each year [9]. Thus a conservative estimate of the annual rate of ABS in USA may be around 7000–14000 cases [5]. As per review, ABS is more common in postmenopausal females in comparison to males. As stated earlier, most of the symptoms are preceded by emotional stressors, which can be emotional or physical. Emotional triggers include death or illness of loved ones, argument, financial loss, and armed robbery. Physical triggers include exercise, swimming, alcohol withdrawal, opiate withdrawal, cocaine use, stress test, severe pain, and noncardiac surgeries. Though the pathophysiology of ABS is incompletely understood, the odds of catecholamines playing a role in triggering the syndrome are greater. Animal models have shown that catecholamine can induce myocardial stunning [10, 11]. Catecholamine and subsequent myocardial insults are also associated with other medical conditions such as SAH/stroke/pheochromocytoma. There is increasing awareness of the interaction between critical brain activity and the heart. ABS clinically presents as ACS; the most common symptom is chest pain. The patient may present with dyspnea/hypotension secondary to decreased stroke volume. Syncope and out-of-hospital arrest are rare presentations. The patient may vary from mildly symptomatic to critically ill, and as many as 20% may require intra-aortic balloon pump or inotropic support. After the history and physical, the next best approach would be getting an EKG. The majority of patients (>80% of patients) have dynamic EKG changes with precordial ST segment elevation or typically diffuse T-wave inversions. EKG changes are usually associated with a modest rise in troponin levels. Echo/Doppler is the next modality to check wall-motion abnormalities; the apical segment is predisposed to stunning in comparison to the base. The explanation for apical involvement and basal sparing is unknown but may be related to increased apical myocardial sensitivity to sympathetic stimulation or to an increased density of catecholamine-sensitive receptors in this region [12, 13]. Functional mitral regurgitation is present in 20% of cases. BNP levels and correlation with the severity of left ventricular function or recovery remain to be validated. Coronary angiography would be the gold standard to differentiate from MI, and as indicated by the review, it is mostly inconsistent with EKG and Echo/Doppler findings. The presentation of ABS mimics ACS/STEMI. The definitive therapy remains to be identified. Initial therapy is directed according to the guidelines of AHA/ACC for acute coronary syndrome and ASA/beta-blocker/nitrates to begin with. ACEI would be beneficial in patients with low-ejection fractions. Assuming there may be platelet hyperaggregability, drugs are often used against platelet activation.

CHF is the most common complication, occurring in approximately 20% of patients, and it is more likely in the presence of right ventricular involvement. Diuretics and vasodilators are effective in these cases. Cardiogenic shock with pump failure is also a complication and is usually treated with balloon counter pulsation. Anticoagulation may be considered in patients with unresolved hypokinesis to prevent thromboembolism. The short-term and long-term prognosis of patients is favorable. Overall, the long-term survival is similar to that of general age-matched population [14]. In-hospital mortality ranges from 0 to 8%. In view of associated mortality, general outcome is slightly worse as compared to normal population, but really better as compared to patients with MI. Recurrence of this syndrome is rare and infrequent. In the largest series so far, which consisted of 88 patients, a recurrence rate of 2.7% has been reported [6]. Our case is one of the unique cases of recurrence after a span of 4 years. One may speculate a genetic predisposition towards developing such a recurrent syndrome. Japanese investigators have detected CD36 deficiency in patients who developed ABS, suggesting that certain genetic profiles are more susceptible [15].

## 4. Conclusion

The case report depicts a rare case of recurrent ABS; only a few case reports have been reported so far. The vulnerability of individuals to physical and emotional stress and the role of genetic susceptibility need to be investigated at the molecular level. ABS is a reversible syndrome predominant in postmenopausal females and is hypothesized to result from a catecholamine surge. Though males have greater adrenergic response to mental stress than females, the protective effect of estrogen prevents the unmasking of symptoms. Estrogen confers cardioprotection in premenopausal women by downregulating the beta-(1)-adrenoceptor and suppressing the expression and activity of protein kinase A. The issue should be elucidated by further information obtained at the molecular level.
